# Construction of Robust Electrothermal Superhydrophobic Surface via Femtosecond Laser for Anti-Icing and Deicing

**DOI:** 10.3390/molecules30081741

**Published:** 2025-04-13

**Authors:** Xuqiao Peng, Daqing Tian, Jingyang Li, Wenxuan Li, Ruisong Jiang, Chaolang Chen

**Affiliations:** School of Mechanical Engineering, Sichuan University, Chengdu 610065, China

**Keywords:** electrothermal superhydrophobic surface, anti-/deicing, femtosecond laser, Ti_3_C_2_ MXene, armor structure, mechanical durability

## Abstract

Electrothermal superhydrophobic surfaces are regarded as possessing significant potential in anti-icing applications. However, their limited mechanical durability has constrained practical implementation. Herein, this work fabricated a robust electrothermal superhydrophobic surface by femtosecond laser texturing combined with the filling of functional coatings of Ti_3_C_2_ MXene and hydrophobic SiO_2_ nanoparticles (modified with dimethyldichlorosilane), which shows great superhydrophobic anti-icing and electrothermal deicing properties, as well as outstanding mechanical durability. The as-prepared electrothermal superhydrophobic surface exhibited a water contact angle of 160.3° and achieved temperature elevation to 104.2 °C within 180 s under an applied voltage of 5 V. Furthermore, the as-prepared electrothermal superhydrophobic surface demonstrated exceptional anti-icing/deicing performance: ice formation time was prolonged to 75.2 s at −35 °C, ice adhesion strength was reduced to 14.65 kPa, and the frozen droplet on the surface melted rapidly within 10.12 s upon electrifying. Moreover, benefiting from the protection of the designed bionic armor structure (honeycomb-like structure), the as-prepared electrothermal superhydrophobic surface maintained outstanding electrothermal and anti-/deicing properties even after 200 times of blade abrasion. This work paves the way for designing robust electrothermal superhydrophobic surfaces in anti-/deicing applications.

## 1. Introduction

Ice formation can cause severe impacts and damage to industrial facilities [1,2,3,4,5]. Traditional deicing methods, such as mechanical deicing and chemical deicing, suffer from drawbacks, including complex equipment, high energy consumption, environmental harm, and low efficiency [6,7]. Consequently, there is an urgent need to develop advanced and efficient anti-icing/deicing technologies.

In recent years, superhydrophobic surfaces have been recognized as having significant potential in the field of anti-icing, which can delay ice formation and reduce ice adhesion strength [8,9,10,11,12,13,14]. Rodič et al. prepared a superhydrophobic surface on the aluminum substrate using laser texturing and grafting techniques, which can significantly delay icing [15]. However, superhydrophobic surfaces alone cannot entirely prevent the accumulation of ice layers [16,17,18]. Many researchers have proposed combining electrothermal deicing with superhydrophobic anti-icing properties to develop multifunctional electrothermal superhydrophobic surfaces [19,20,21]. Li et al. fabricated an electrothermal superhydrophobic surface using silver nanowires and epoxy resin, which exhibited a contact angle of 162.3° [22]. Under a power density of 0.25 W/cm^2^, the surface temperature increased from 20 °C to 108.7 °C, and the ice formation time was extended from 724 s to 936 s. Despite significant progress in the research of electrothermal superhydrophobic surfaces, their practical application is often limited by mechanical abrasion caused by external forces [23,24,25]. The surface properties could be degraded or even completely lost after abrasion, thereby restricting the applications of electrothermal superhydrophobic surfaces [26,27]. Consequently, enhancing the mechanical durability of electrothermal superhydrophobic surfaces is an urgent and critical need. Wang et al. have prepared an armor structure for the first time and demonstrated that it can effectively improve the mechanical durability and robustness of the superhydrophobic surface [28]. This inspired researchers to improve the mechanical durability of electrothermal superhydrophobic surfaces by designing suitable armor structures.

**Figure 1:**
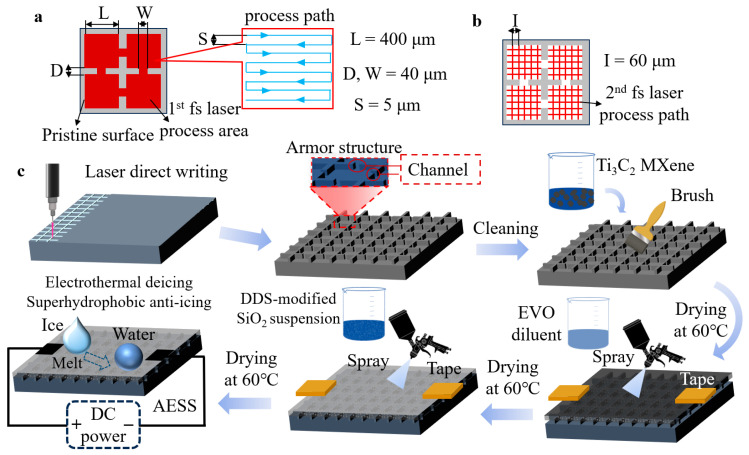
(**a**) Schematic diagram of the first laser scanning pattern. (**b**) Schematic diagram of the second laser scanning pattern. (**c**) Schematic diagram of the preparation of AESS.

Here, we fabricated a robust armor-protected electrothermal superhydrophobic surface (AESS). As shown in Figure 1 and Figure S1, first, the bionic armor structure was constructed on the substrate (taking a silicon wafer as an example) by femtosecond laser texturing. Subsequently, the laser-textured surface was filled with functional coatings of Ti_3_C_2_ MXene and hydrophobic SiO_2_ nanoparticles to obtain AESS. Ti_3_C_2_ MXene has attracted significant interest due to its exceptionally high theoretical thermal conductivity and electrical conductivity, positioning it as an excellent alternative to carbon nanotubes, graphene, and similar materials, which makes Ti_3_C_2_ MXene highly promising for applications in the field of deicing [29,30,31]. Therefore, the Ti_3_C_2_ MXene was selected as a representative of electrothermal materials to endow the surface with electrothermal properties. Meanwhile, the fluorine-free dimethyldichlorosilane (DDS)-modified SiO_2_ nanoparticles were selected as a hydrophobic paint to endow the surface with superhydrophobic properties. The electrothermal performance, wettability, mechanical durability, and anti-/deicing performance of the as-prepared AESS were systematically studied.

## 2. Results and Discussion

### 2.1. Morphology and Chemical Component Characterization of AESS

**Figure 2:**
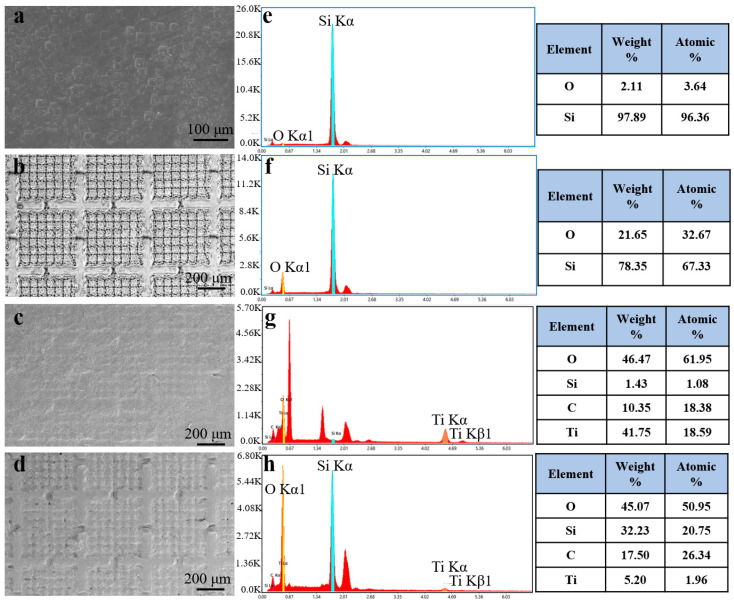
SEM images of (**a**) the silicon wafer, (**b**) laser-textured silicon wafer, (**c**) laser-textured silicon wafer with Ti_3_C_2_ MXene coating, and (**d**) laser-textured silicon wafer with Ti_3_C_2_ MXene and hydrophobic SiO_2_ coating. EDS of (**e**) the silicon wafer, (**f**) laser-textured silicon wafer, (**g**) laser-textured silicon wafer with Ti_3_C_2_ MXene coating, and (**h**) laser-textured silicon wafer with Ti_3_C_2_ MXene and hydrophobic SiO_2_ coatings.

As shown in Figure 2a, the surface of the pristine silicon wafer is smooth. After laser texturing, uniformly distributed armor structures appeared on the surface, and four channels existed around each armor structure (Figure 2b). After the laser-textured surface was coated with Ti_3_C_2_ MXene, the armor structure was filled (Figure 2c). Then, the surface became rougher after being coated with the hydrophobic SiO_2_ nanoparticles modified with DDS (Figure 2d).

EDS was employed to analyze the element distribution of the samples at each preparation stage. The pristine silicon wafer had a Si atomic percentage of 96.36%, as shown in Figure 2e. After laser texturing, the Si atomic percentage decreased to 67.33%, while the O atomic percentage increased from 3.64% to 32.67% (Figure 2f), which was caused by the oxidation of surface silicon atoms during the laser texturing process [32]. As shown in Figure 2g, after being filled with Ti_3_C_2_ MXene, the atomic percentage of Si dropped to 1.08%, while the atomic fractions of C and Ti increased from 0% to 18.38% and 18.59%, respectively. It can be concluded that the Ti_3_C_2_ MXene coating has been successfully prepared on the surface (Figure S2a). After being coated with hydrophobic SiO_2_ nanoparticles, the atomic percentage of the Si and C elements increased to 20.75% and 26.34%, respectively, and the Ti element decreased to 1.96% (Figure 2h). At the same time, the Si element was evenly distributed on the surface (Figure S2b). This shows that after being sprayed with hydrophobic SiO_2_ nanoparticles, the distribution of SiO_2_ nanoparticles on AESS was very uniform, and the original Ti_3_C_2_ MXene coating was covered with the hydrophobic SiO_2_ nanoparticles.

### 2.2. Characterization of the Wettability and Electrothermal Properties of AESS

**Figure 3:**
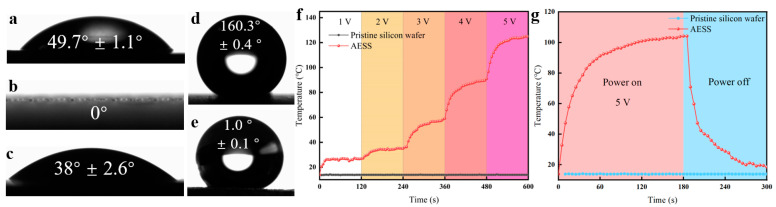
(**a**) WCA of the pristine silicon wafer. (**b**) WCA of the laser-textured silicon wafer. (**c**) WCA of the laser-textured silicon wafer after being filled with Ti_3_C_2_ MXene. (**d**) WCA of AESS. (**e**) Roll-off angle of AESS. (**f**) Temperature of the pristine silicon wafer and AESS at different input voltages at different times. (**g**) Temperature of the pristine silicon wafer and AESS at an input voltage of 5 V at different times.

As shown in Figure 3a–e, the WCA of the pristine silicon wafer and the laser-textured silicon wafer are 49.7° and 0°, respectively. It is shown that the pristine silicon wafer is highly hydrophilic. After laser texturing, the hydrophilicity was further enhanced. After being filled with Ti_3_C_2_ MXene, the WCA increased to 38°. After being modified with hydrophobic SiO_2_, the WCA of the as-prepared AESS was further increased to 160.3°, and the roll-off angle of AESS is 1°. This indicates that the as-prepared AESS is a superhydrophobic surface. This is because AESS was modified by hydrophobic SiO_2_ nanoparticles, and the wetting state changed from the Wenzel state to the Cassie state, thus enhancing hydrophobicity [33,34,35,36].

To explore the electrothermal properties of AESS, the pristine silicon wafer and AESS were supplied with the input voltage. As shown in Figure 3f, when the voltage was increased from 1 V to 5 V, the temperature of the pristine silicon wafer did not change, and the surface temperature was consistent with the ambient temperature. However, the temperature of AESS was increased with the increase in input voltage; the maximum temperatures of AESS reached 27.3 °C, 35.2 °C, 59.1 °C, 90.9 °C, and 125 °C, respectively. As shown in Figure 3g, the temperature of the pristine silicon wafer was not changed within 300 s when the input voltage was maintained at 5 V (the power density is 1.25 W/cm^2^). As a contrast, the temperature of AESS rose rapidly from 13.9 °C to 92.5 °C within 60 s and reached 104.2 °C at 180 s. This shows that AESS has excellent electrothermal performance and a fast electrothermal response rate. This is because of the low resistivity and high thermal conductivity of the filled Ti_3_C_2_ MXene, which improves the electrothermal properties of the surface.

### 2.3. Anti-Icing and Deicing Performance of AESS

**Figure 4:**
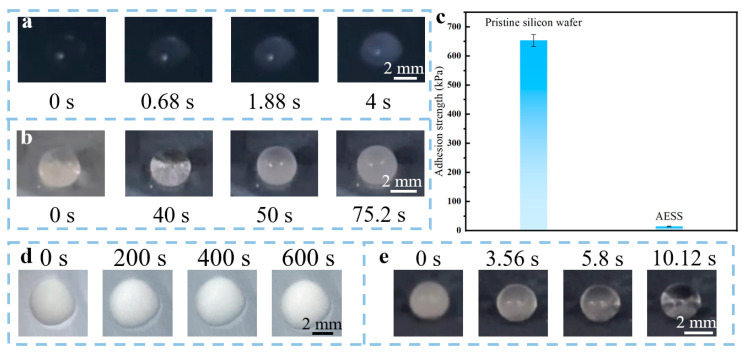
Freezing process of water droplet (10 μL) on (**a**) the pristine silicon wafer and (**b**) AESS at −35 °C. (**c**) The adhesion strength of ice on the pristine silicon wafer and AESS. Melting process of ice (frozen by 10 μL water) on (**d**) the pristine silicon wafer and (**e**) AESS at −35 °C with an input voltage of 5 V.

To study the anti-icing performance of AESS, the icing process of a water droplet (10 μL) on the pristine silicon wafer and AESS was observed. As illustrated in Figure 4a, the droplet froze very quickly on the pristine silicon wafer; the freezing time was only about 4 s (the average freezing time is 3.96 ± 0.19 s). In contrast, as illustrated in Figure 4b, the freezing process of droplets on AESS was very slow; the droplet was completely frozen at 75.2 s (the average freezing time is 75.91 ± 4.72 s), and its freezing time was extended by 18.8 times compared with the pristine silicon wafer. The adhesion strength of the ice (frozen by 1 mL of water) on the pristine silicon wafer and AESS was also tested. As shown in Figure 4c, the ice adhesion strength of the pristine silicon wafer is 653.84 kPa. However, the ice adhesion strength of AESS is only 14.65 kPa, which is only 2.24% of that of the pristine silicon wafer. It is shown that AESS can tremendously reduce the ice adhesion strength. This is because the air preserved in the hydrophobic coating of AESS can reduce the heat exchange rate and the contact area between the droplet and the surface, endowing AESS with excellent anti-icing performance.

To study the active deicing performance of AESS, the pristine silicon wafer and AESS were placed in a low-temperature environment (−35 ± 2 °C) and supplied with an input voltage of 5 V to observe the melting process of ice (frozen by 10 μL of water) on different surfaces. As shown in Figure 4d, after 600 s, the appearance of the frozen droplets on the pristine silicon wafer was not changed, indicating that the frozen droplet was not melted. However, as shown in Figure 4e, the ice on AESS was completely melted just at 10.12 s (the average melting time is 11.12 ± 0.95 s), demonstrating outstanding deicing performance. In addition, the anti-frosting performance was also studied by recording the surface appearance changes in AESS under high humidity and low-temperature conditions. As shown in Figure S3, AESS was completely covered by frost at 75.6 s (the average frosting time is 73.76 ± 6.29 s). After being supplied with a voltage of 5 V (power density of 1.25 W/cm^2^), the frost was completely melted at 7.36 s (the average melting time is 7.09 ± 0.95 s), demonstrating excellent anti-frosting and defrosting performance. This is because AESS has favorable electrothermal properties; the heat generated on its surface when the electricity is applied is enough to rapidly melt ice at a low temperature.

### 2.4. Mechanical Durability of As-Prepared AESS

**Figure 5:**
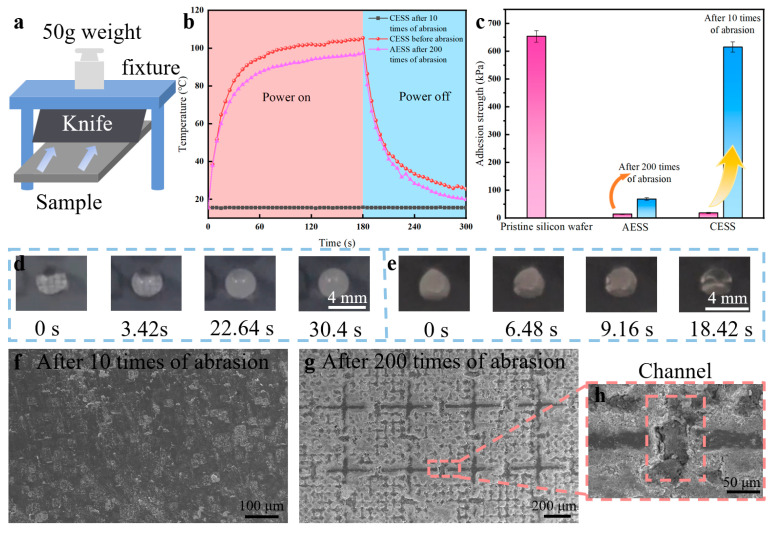
(**a**) Schematic diagram of the knife abrasion test. (**b**) The temperature of different samples at an input voltage of 5 V at different times. (**c**) The ice adhesion strength of different samples. (**d**) Freezing process of a water droplet on AESS after 200 times of abrasion at −35 °C. (**e**) Melting process of the frozen droplet on AESS after 200 times of abrasion at −35 °C with an input voltage of 5 V. (**f**) SEM images of CESS after 10 times of abrasion. (**g**) SEM images of AESS after 200 times of abrasion. (**h**) SEM images of the channel structure of AESS after 200 times of abrasion.

To investigate the mechanical durability of AESS, the blade abrasion test was conducted on the pristine silicon wafer, the common electrothermal superhydrophobic surface (CESS: pristine silicon wafer directly covered with the as-prepared hydrophobic SiO_2_ and Ti_3_C_2_ MXene coating), and AESS. As illustrated in Figure 5a, the knife is fixed in the fixture, and a 50 g weight is placed above the fixture. The blade was positioned vertically in contact with the sample surface, and then the sample was moved by a linear guide at a speed of 1 mm/s until the blade had fully traversed the surface. This process was defined as one cycle of blade abrasion test. The WCA of CESS decreased remarkably from 153° to 41.4° after just 10 times of abrasion (Figure S4a,b). However, the WCA of AESS still reached 134.2° after 200 times of abrasion (Figure S4c). This indicates that the hydrophobicity of AESS is well maintained after 200 times of abrasion. As shown in Figure 5b, the temperature of CESS increased from 15.6 °C to 105.4 °C within 180 s at an input voltage of 5 V. However, after 10 times of abrasions, the temperature of CESS was kept unchanged near 15.6 °C as time increased, indicating that the electrothermal performance was completely lost. The temperature of the as-prepared AESS rose rapidly from 15.6 °C to 88.2 °C within 60 s and reached 97.3 °C at 180 s. This shows that AESS still has excellent electrothermal properties after 200 times of abrasion.

The mechanical durability of the anti-/deicing properties of AESS was also studied. As shown in Figure 5c, the ice adhesion strength of CESS increased sharply from 18.71 kPa to 615.24 kPa after only 10 times of abrasion, close to that of the pristine silicon wafer. However, the ice adhesion strength of AESS increased slightly from 14.65 kPa to 68.91 kPa after 200 times of abrasion. As illustrated in Figure 5d, the droplet was frozen at 30.4 s at −35 °C on AESS after 200 times of abrasion (the average freezing time is 28.61 ± 1.3 s). This shows that AESS still has excellent anti-icing properties after 200 times of abrasion because its hydrophobic properties are well preserved. As illustrated in Figure 5e, the frozen droplet was melted at 18.42 s at −35 °C on AESS after 200 times of abrasion with an input voltage of 5 V (the average melting time is 18.87 ± 0.62 s). The deicing time is only 8.3 s longer than that of AESS before abrasion, indicating that AESS still has efficient deicing performance after abrasion.

Further, the morphologies of different samples after abrasion were analyzed. As shown in Figure 5f, after 10 times of abrasion, the surface morphology of the CESS became similar to that of the pristine silicon wafer, indicating that the electrothermal superhydrophobic functional coating on its surface has been fully removed. As a result, the hydrophobic and electrothermal properties of CESS were lost after abrasion. As shown in Figure 5g, after 200 times of abrasion, the coating on the frame of the armor structure of AESS was removed and the armor structure became more prominent. However, the coating inside the armor structure is intact, maintaining AESS’s hydrophobic properties. In addition, the coating inside the channel was also completely protected after abrasion (Figure 5h), so that AESS could still form a complete conductive circuit, maintaining the high conductivity and electrothermal property of AESS. This shows that the as-prepared armor structure can withstand abrasion instead of the fragile coating inside it, effectively improving the mechanical durability of the sample.

As shown in Table S1, compared to recently reported laser-textured superhydrophobic surfaces [12,15,37,38,39]. The as-prepared AESS demonstrated better anti-icing and electrothermal properties, and the mechanical durability has also been validated.

## 3. Materials and Methods

### 3.1. Materials

Silicon wafer (20 × 20 × 0.6 mm) was bought from Kaihua Lijing Electronics Co., Ltd., China (Quzhou, China). Ti_3_C_2_ MXene suspension was purchased from Foshan Xinxi Technology Co., Ltd., China (Foshan, China). Absolute ethanol and hydrophobic fumed silicon dioxide nanoparticles (modified with dimethyldichlorosilane (DDS), AEROSIL R972) were bought from Shanghai Energy Biochemical Co., Ltd., China (Shanghai, China). Tape was bought from Dongguan Xinshi packaging materials Co., Ltd., China (Dongguan China). The distilled water was purchased from A.S.WATSON TM LIMITED (Chengdu, China). Evo-stik serious glue was bought from TB.com on 16 November 2023. The knife was bought from Deli Group Limited (Chengdu, China).

### 3.2. Preparation of AESS

The armor structure with channels was prepared on the substrate by two rounds of the fs laser process. In the first laser processing, the length and width of the individual armor structure are designed at 400 μm, the length and width of the channel are 40 μm, and inside the armor structure, the interval of the laser scanning paths is 5 μm. The fs laser has a repetition of 300 kHz, a wavelength of 1030 nm, an output power of 7 W, and a pulse duration of 300 fs, the scanning speed was kept at 500 mm/s. The scanning process was repeated 10 times. In the second laser processing, the mastoid structure inside the armor structure is generated by X-Y cross-scanning, and the interval of the laser scanning paths is 60 μm. The output power was increased to 9 W, the scanning speed was increased to 1000 mm/s, and the scanning times were increased to 20. Then, the sample was immersed in absolute ethanol and ultrasonically cleaned for 5 min to remove fragments. Then, the Ti_3_C_2_ MXene suspension was poured on the surface of the sample, brushed evenly, and placed in a drying oven at 60 °C for 30 min. The brush dry steps were repeated 5 times to fill the armor structure. A 2 mm × 2 mm area on both sides of the sample was masked by tape to ensure the conductivity of the sample. A total of 1 g EVO-stik serious glue was added to 99 g absolute ethanol and stirred for 10 min to obtain EVO-diluent. The distance between the spray gun and the sample was 25 cm. The sample was evenly sprayed with EVO-diluent and then dried at 60 °C for 10 min. A total of 3 g DDS-modified SiO_2_ nanoparticles were added to 97 g of absolute ethanol and followed by magnetic stirring for 30 min to obtain a SiO_2_ suspension. The sample was evenly sprayed with SiO_2_ suspension and then dried at 60 °C for 30 min. Finally, the tape was removed to obtain AESS.

### 3.3. Characterizations and Measurements

The surface morphology of different samples was observed by scanning electron microscope (SEM, Apreo, Chengdu, China). An energy-dispersive spectroscopy (EDS, Bruker, Chengdu, China) analysis was used to determine the element distribution of different samples. The water contact angle (WCA) was measured by a dynamic contact angle measuring instrument (Dataphysics, OCA15, Berlin, Germany). A 3 μL water droplet was dropped on the sample surface. Optical photos of the water droplet were recorded using a high-speed camera and the image was analyzed to obtain WCA. A 10 μL water droplet was used to measure the roll-off angle. WCA and SA were measured 3 times and averaged.

A refrigerator (BD/BC-48A108B, Huabao, Shenzhen, China) was used to build a low-temperature environment for icing (inside temperature = −35 ± 2 °C, inside relative humidity = 30 ± 5%). A DC power (HY3005B, Huayi Electronic Industry Co., Ltd., China, Yixing, China) was used to apply voltage to the samples and an infrared imager (Uti120S, UNI-trend Technology Co., Ltd., China, Dongguan, China) was used to record the temperature of the samples. The ambient temperature was 15 ± 2 °C and the relative humidity was 45 ± 5%.

The ice adhesion strength measurement experiment was carried out at an ambient temperature measured three times and averaged. The ice adhesion strength experiment is described as follows: a cylindrical tube was prepared by 3D printing (P1P, Bambu lab, Shenzhen, China) and hydrophobically modified. A total of 1 mL of distilled water was injected into the hydrophobic 3D-printed cylindrical tube (base area = 1 cm^2^) and frozen in the refrigerator for 3 h. The radius of the force sensor probe is 7 mm, and the distance between the bottom of the probe and the sample surface in the vertical direction is 1 mm. Then, the tube was pushed by a force sensor fixed on a lead rail with a speed of 100 μm/s. The thrust the sensor receives during the push is recorded. The adhesion strength is described as follows [40,41,42]:

τ = F/S(1)

where τ is the adhesion strength, F is the maximum thrust, and S is the contact area between the ice and the surface.

The anti-frosting experiment is described as follows: the sample was placed in the refrigerator. A humidifier (Hulker) was used to raise the humidity inside the refrigerator to 70%.

The active deicing process is described as follows: 10 μL water droplet was placed on the surface of the sample and frozen in a refrigerator for 3 h. A smartphone (iPhone 15) was used to record the morphologic changes in the frozen droplet after the sample was electrified.

## 4. Conclusions

In summary, this work constructed a robust armor-protected electrothermal superhydrophobic surface for anti-/deicing, which has high abrasion resistance. A bionic armor structure is created on the surface by using a femtosecond laser, then filled with Ti_3_C_2_ MXene particles through a brushing technique, followed by spraying with fluorine-free DDS-modified hydrophobic SiO_2_ nanoparticles. Through the analysis of experimental phenomena, it was found that the as-prepared AESS is endowed with excellent anti-icing/deicing properties and mechanical durability. Compared with the pristine silicon wafer, the AESS demonstrated significant performance enhancement: WCA was increased from 49.7° to 160.3°, icing time was delayed from 4 s to 75.2 s, and ice adhesion strength was decreased from 653.84 kPa to 14.65 kPa. When AESS was subjected to a 5 V input voltage, the AESS achieved a rapid thermal response, the temperature increased from 13.9 °C to 104.2 °C within 180 s and enabled complete melting of the surface-adhered frozen droplet within 10.12 s. This verifies the electrothermal deicing and superhydrophobic anti-icing properties of AESS. Moreover, under the effect of the as-prepared armor structure, AESS can still maintain excellent anti-/deicing properties after 200 times of blade abrasion. This provides new ideas for designing robust anti-icing surfaces.

## Data Availability

The original contributions presented in this study are included in the article/Supplementary Materials. Further inquiries can be directed to the corresponding author.

## Supplementary Materials

The following supporting information can be downloaded at: https://www.mdpi.com/article/10.3390/molecules30081741/s1, Figure S1: Schematic diagram of the femtosecond laser system; Figure S2: (a) Element distribution map of Ti element of the laser-textured sample after being filled with Ti_3_C_2_ MXene. (b) Element distribution map of Si element of AESS; Figure S3: (a) Frosting and (b) defrosting process of AESS; Figure S4: WCA of CESS (a) before and (b) after 10 times of sandpaper abrasion; Table S1: Comparison between this work and other reports.

## Abbreviations

The following abbreviations are used in this manuscript:

AESS	Armor-protected electrothermal superhydrophobic surface
CESS	Common electrothermal superhydrophobic surface
SEM	Scanning electron microscope
EDS	Energy-dispersive spectroscopy
WCA	Water contact angle
DDS	dimethyldichlorosilane

## References

[1] Andenæs E., Jelle B.P., Ramlo K., Kolås T., Selj J., Foss S.E. (2018). The influence of snow and ice coverage on the energy generation from photovoltaic solar cells. Sol. Energy.

[2] Matejicka L., Georgakis C.T. (2022). A review of ice and snow risk mitigation and control measures for bridge cables. Cold Reg. Sci. Technol..

[3] Chen C., Tian Z., Luo X., Jiang G., Hu X., Wang L., Peng R., Zhang H., Zhong M. (2022). Micro–Nano-Nanowire Triple Structure-Held PDMS Superhydrophobic Surfaces for Robust Ultra-Long-Term Icephobic Performance. ACS Appl. Mater. Interfaces.

[4] He H., Guo Z. (2021). Superhydrophobic materials used for anti-icing Theory, application, and development. iScience.

[5] Cao Y., Tan W., Wu Z. (2018). Aircraft icing: An ongoing threat to aviation safety. Aerosp. Sci. Technol..

[6] Wang T., Zheng Y.H., Raji A.R.O., Li Y.L., Sikkema W.K.A., Tour J.M. (2016). Passive Anti-Icing and Active Deicing Films. ACS Appl. Mater. Interfaces.

[7] Farzaneh M., Ryerson C.C. (2011). Anti-icing and deicing techniques. Cold Reg. Sci. Technol..

[8] Wang L., Tian Z., Jiang G., Luo X., Chen C., Hu X., Zhang H., Zhong M. (2022). Spontaneous dewetting transitions of droplets during icing & melting cycle. Nat. Commun..

[9] Zhao W., Xiao L., He X., Cui Z., Fang J., Zhang C., Li X., Li G., Zhong L., Zhang Y. (2021). Moth-eye-inspired texturing surfaces enabled self-cleaning aluminum to achieve photothermal anti-icing. Opt. Laser Technol..

[10] Luo X.T., Li C.J. (2019). Bioinspired Mechanically Robust Metal-Based Water Repellent Surface Enabled by Scalable Construction of a Flexible Coral-Reef-Like Architecture. Small.

[11] Guo Q., Ma J., Yin T., Jin H., Zheng J., Gao H. (2024). Superhydrophobic Non-Metallic Surfaces with Multiscale Nano/Micro-Structure: Fabrication and Application. Molecules.

[12] Kovač N., Može M., Kapun B., Golobič I., Milošev I., Rodič P. (2025). Enhanced corrosion resistance and self-cleaning of AlSi7Mg0.3 via superhydrophobic surface using laser structuring and stearic acid grafting. Surf. Interfaces.

[13] Yamada Y., Onishi G., Horibe A. (2019). Sessile Droplet Freezing on Hydrophobic Structured Surfaces under Cold Ambient Conditions. Langmuir.

[14] Boinovich L.B., Emelyanenko A.M. (2024). Recent progress in understanding the anti-icing behavior of materials. Adv. Colloid Interface Sci..

[15] Rodič P., Kovač N., Kralj S., Jereb S., Golobič I., Može M., Milošev I. (2024). Anti-corrosion and anti-icing properties of superhydrophobic laser-textured aluminum surfaces. Surf. Coat. Technol..

[16] Wei X., Cai F., Wang J. (2023). Electrothermal/photothermal superhydrophobic coatings based on micro/nano graphite flakes for efficient anti-icing and de-icing. Prog. Org. Coat..

[17] Fan J., Long Z., Wu J., Gao P., Wu Y., Si P., Zhang D. (2023). Electrothermal superhydrophobic epoxy nanocomposite coating for anti-icing/deicing. J. Coat. Technol. Res..

[18] Jiang L., Sun J., Lin Y., Gong M., Tu K., Chen Y., Xiao T., Xiang P., Tan X. (2024). The preparation of CNTs/GP/TiN/PDMS/PVDF superhydrophobic coating with strong photothermal and electrothermal properties for anti-icing and de-icing. Surf. Coat. Technol..

[19] Li D., Zhang Y., Yuan L., Ding Z., Liang G., Gu A. (2020). Superhydrophobic and self-healable tri-layered composites with great thermal resistance and electrothermal ability. Compos. Commun..

[20] Zhao Z., Chen H., Zhu Y., Liu X., Wang Z., Chen J. (2022). A robust superhydrophobic anti-icing/de-icing composite coating with electrothermal and auxiliary photothermal performances. Compos. Sci. Technol..

[21] Yang C., Ji H.Z., Song L.H., Su H.X., Qi Z.P., Wang Y., Cheng E., Zhao L.B., Hu N. (2024). Multifunctional superhydrophobic composite film with icing monitoring and anti-icing/deicing performance. Compos. Sci. Technol..

[22] Li K., Wang Q., Zhou X., He Y., Shi Y., Qin M., Wu B., Chen N., Liu R., Yi X. (2024). Electrothermal/Superhydrophobic Anti-Deicing Coating with a Sandwich Structure Based on Micro-Nanomaterials. ACS Appl. Nano Mater..

[23] Tenjimbayashi M., Samitsu S., Naito M. (2019). Simultaneous Detection and Repair of Wetting Defects in Superhydrophobic Coatings via Cassie-Wenzel Transitions of Liquid Marbles. Adv. Funct. Mater..

[24] Li K., Wang Y.S., Jiang Z.L., Wong H., Zhou T., Wu J.X., Zhang J.H., Zhang A.M. (2022). Functional building devices using laser-induced selective metallization on magnesium oxychloride cement composites. Cem. Concr. Compos..

[25] Huang B., Jiang S., Diao Y., Liu X., Liu W., Chen J., Yang H. (2020). Hydrogels as Durable Anti-Icing Coatings Inhibit and Delay Ice Nucleation. Molecules.

[26] Yan X., Chen F., Sett S., Chavan S., Li H., Feng L., Li L., Zhao F., Zhao C., Huang Z. (2019). Hierarchical Condensation. ACS Nano.

[27] Li X., Su H., Li H., Tan X., Lin X., Wu Y., Xiong X., Li Z., Jiang L., Xiao T. (2024). Photothermal superhydrophobic surface with good corrosion resistance, anti-/de-icing property and mechanical robustness fabricated via multiple-pulse laser ablation. Appl. Surf. Sci..

[28] Wang D., Sun Q., Hokkanen M.J., Zhang C., Lin F.-Y., Liu Q., Zhu S.-P., Zhou T., Chang Q., He B. (2020). Design of robust superhydrophobic surfaces. Nature.

[29] Shahzad F., Alhabeb M., Hatter C.B., Anasori B., Man Hong S., Koo C.M., Gogotsi Y. (2016). Electromagnetic interference shielding with 2D transition metal carbides (MXenes). Science.

[30] Zhou B., Zhang Z., Li Y., Han G., Feng Y., Wang B., Zhang D., Ma J., Liu C. (2020). Flexible, Robust, and Multifunctional Electromagnetic Interference Shielding Film with Alternating Cellulose Nanofiber and MXene Layers. ACS Appl. Mater. Interfaces.

[31] Alhabeb M., Maleski K., Anasori B., Lelyukh P., Clark L., Sin S., Gogotsi Y. (2017). Guidelines for Synthesis and Processing of Two-Dimensional Titanium Carbide (Ti3C2Tx MXene). Chem. Mater..

[32] Zhang D., Liu R., Li Z. (2022). Irregular LIPSS produced on metals by single linearly polarized femtosecond laser. Int. J. Extrem. Manuf..

[33] Pan R., Cai M., Liu W., Luo X., Chen C., Zhang H., Zhong M. (2019). Extremely high Cassie–Baxter state stability of superhydrophobic surfaces via precisely tunable dual-scale and triple-scale micro–nano structures. J. Mater. Chem. A.

[34] Tang B.-H., Wang Q., Han X.-C., Zhou H., Yan X.-J., Yu Y., Han D.-D. (2022). Fabrication of anti-icing/de-icing surfaces by femtosecond laser. Front. Chem..

[35] Cui T., Zheng Y., Hu M., Lin B., Wang J., Cai W., Fei B., Zhu J., Hu Y. (2024). Biomimetic Multifunctional Graphene-Based Coating for Thermal Management, Solar De-Icing, and Fire Safety: Inspired from the Antireflection Nanostructure of Compound Eyes. Small.

[36] Marmur A., Della Volpe C., Siboni S., Amirfazli A., Drelich J.W. (2017). Contact angles and wettability: Towards common and accurate terminology. Surf. Innov..

[37] Li S., Zhong M., Zou Y., Xu M., Liu X., Xing X., Zhang H., Jiang Y., Qiu C., Qin W. (2023). Fabrication of Micron-Structured Heatable Graphene Hydrophobic Surfaces for Deicing and Anti-Icing by Laser Direct Writing. Coatings.

[38] Xing W., Li Z., Yang H., Li X., Wang X., Li N. (2019). Anti-icing aluminum alloy surface with multi-level micro-nano textures constructed by picosecond laser. Mater. Des..

[39] Wang H., He M., Liu H., Guan Y. (2019). One-Step Fabrication of Robust Superhydrophobic Steel Surfaces with Mechanical Durability, Thermal Stability, and Anti-icing Function. ACS Appl. Mater. Interfaces.

[40] Xuan S., Yin H., Li G., Zhang Z., Jiao Y., Liao Z., Li J., Liu S., Wang Y., Tang C. (2023). Trifolium repens L.-Like Periodic Micronano Structured Superhydrophobic Surface with Ultralow Ice Adhesion for Efficient Anti-Icing/Deicing. ACS Nano.

[41] Wang W., Chang J., Chen L., Weng D., Yu Y., Hou Y., Yu G., Wang J., Wang X. (2024). A laser-processed micro/nanostructures surface and its photothermal de-icing and self-cleaning performance. J. Colloid Interface Sci..

[42] Maitra T., Jung S., Giger M.E., Kandrical V., Ruesch T., Poulikakos D. (2015). Superhydrophobicity vs. Ice Adhesion: The Quandary of Robust Icephobic Surface Design. Adv. Mater. Interfaces.

