# Supplementary material for: Construction of Robust Electrothermal Superhydrophobic Surface via Femtosecond Laser for Anti-Icing and Deicing
Source: Molecules. 2025 Apr 13;30(8):1741. doi: 10.3390/molecules30081741 (PMC12029293; doi:10.3390/molecules30081741)
Supplement: Supplementary file 1 [file molecules-30-01741-s001.zip › molecules-3530617-supplementary.pdf]

Supporting Information

Construction of robust electrothermal superhydrophobic surface via femtosecond laser for anti-icing and deicing

Xuqiao Peng†, Daqing Tian†, Jingyang Li, Wenxuan Li, Ruisong Jiang, Chaolang Chen*,

School of Mechanical Engineering, Sichuan University, Chengdu, 610065, China.
13687264354@163.com (X.P.); tiandaqing@scu.edu.cn (D.T.); sotvin@163.com (J.L.); liwenxuan2002@gmail.com (W.L.); jiangrs@scu.edu.cn (R.J.)

* Correspondence: chaolangchen@scu.edu.cn

† These authors contributed equally to this work.

Additional Figures

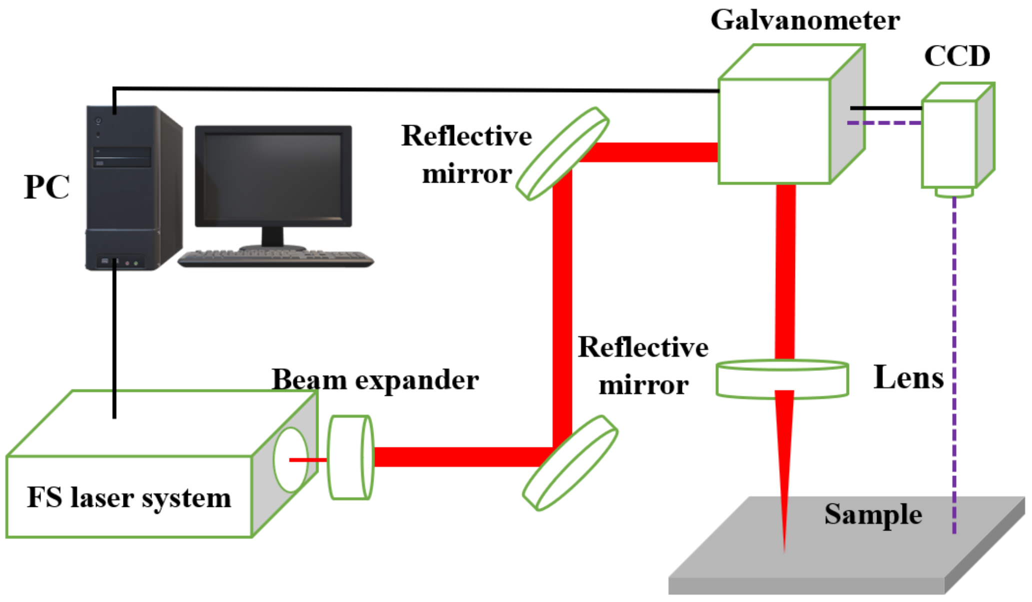

Figure S1. Schematic diagram of the femtosecond laser system.

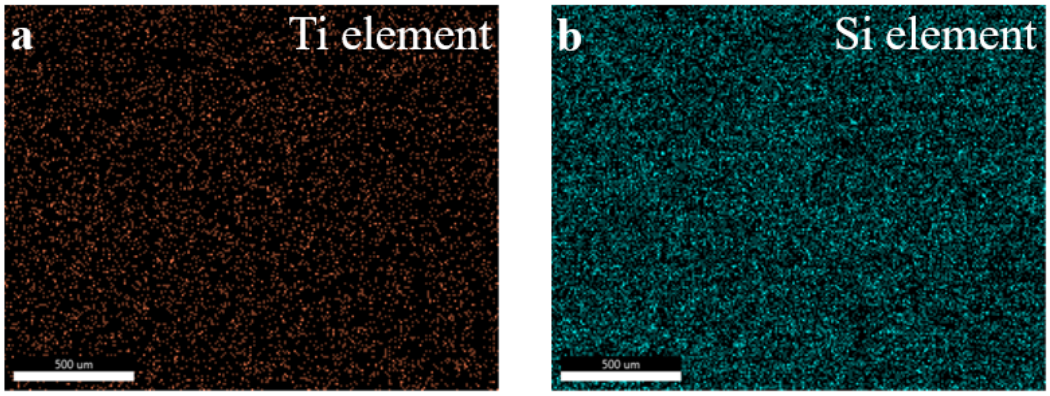

Figure S2. (a) Element distribution map of Ti element of the laser-textured sample after being filled with Ti_3C_2 MXene. (b) Element distribution map of Si element of AESS.

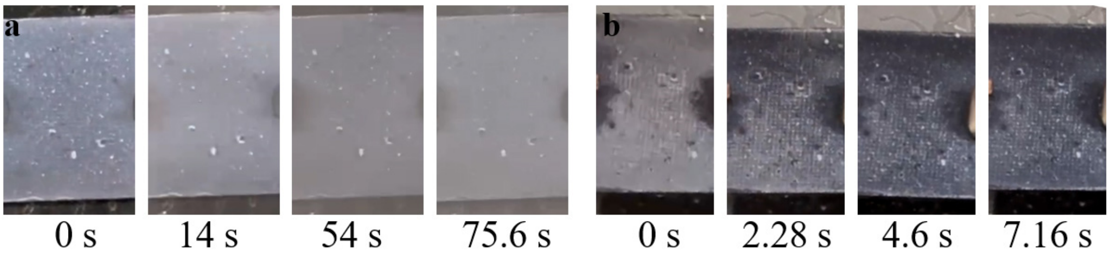

Figure S3. (a) Frosting and (b) defrosting process of AESS.

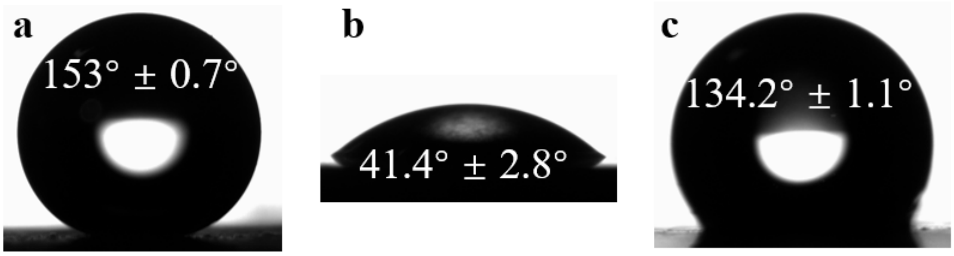

Figure S4. WCA of CESS (a) before and (b) after 10 times of sandpaper abrasion. (b) WCA of AESS after 200 times of abrasion.

Table S1. Comparison between this work and other reports.

**Reference**	**Water contact angle**	**Electrothermal performance**	**Ice adhesion strength**	**Icing delay time**	**Mechanical durability test**
This work	160.2°	90.3℃ (5 V)	14.65 kPa	75.2 s (-35℃)	Yes
Ref 15	155°	No	-	25.2 s (-20℃)	No
Ref 12	153°	No	-	-	No
Ref 37	151°	81.5℃ (5 V)	-	68 s (-23℃)	Yes
Ref 38	168.36°	No	-	53 s (-23℃)	No
Ref 39	159.2°	No	-	No freezing (-8.5℃)	Yes
